# Prediction of the synergistic effect of antimicrobial peptides and antimicrobial agents via supervised machine learning

**DOI:** 10.1186/s42490-024-00075-z

**Published:** 2024-01-17

**Authors:** Basak Olcay, Gizem D. Ozdemir, Mehmet A. Ozdemir, Utku K. Ercan, Onan Guren, Ozan Karaman

**Affiliations:** 1 Department of Biomedical Engineering, Graduate School of Natural and Applied Sciences, Izmir Katip Celebi University, Izmir, Turkey; 2 Department of Biomedical Engineering, Faculty of Engineering and Architecture, Izmir Katip Celebi University, Izmir, Turkey

**Keywords:** Antimicrobial peptides, Antimicrobial agents, Synergistic effect, Fractional inhibitory concentration, Machine learning, Artificial intelligence

## Abstract

**Background:**

Infectious diseases not only cause severe health problems but also burden the healthcare system. Therefore, the effective treatment of those diseases is crucial. Both conventional approaches, such as antimicrobial agents, and novel approaches, like antimicrobial peptides (AMPs), are used to treat infections. However, due to the drawbacks of current approaches, new solutions are still being investigated. One recent approach is the use of AMPs and antimicrobial agents in combination, but determining synergism is with a huge variety of AMPs time-consuming and requires multiple experimental studies. Machine learning (ML) algorithms are widely used to predict biological outcomes, particularly in the field of AMPs, but no previous research reported on predicting the synergistic effects of AMPs and antimicrobial agents.

**Results:**

Several supervised ML models were implemented to accurately predict the synergistic effect of AMPs and antimicrobial agents. The results demonstrated that the hyperparameter-optimized Light Gradient Boosted Machine Classifier (oLGBMC) yielded the best test accuracy of 76.92% for predicting the synergistic effect. Besides, the feature importance analysis reveals that the target microbial species, the minimum inhibitory concentrations (MICs) of the AMP and the antimicrobial agents, and the used antimicrobial agent were the most important features for the prediction of synergistic effect, which aligns with recent experimental studies in the literature.

**Conclusion:**

This study reveals that ML algorithms can predict the synergistic activity of two different antimicrobial agents without the need for complex and time-consuming experimental procedures. The implications support that the ML models may not only reduce the experimental cost but also provide validation of experimental procedures.

**Supplementary Information:**

The online version contains supplementary material available at 10.1186/s42490-024-00075-z.

## Background

One of the most serious disease groups that can arise with acute or prolonged complications and pose a severe threat to human life is infectious diseases [1]. Infectious diseases may have diverse origins, with microorganisms, including opportunistic pathogens residing within the human body, being among the potential causative factors. Under specific shifts in environmental conditions, these opportunistic microorganisms can transition to a pathogenic state, resulting in infections that pose formidable treatment challenges [2]. Additionally, the spectrum of infectious diseases extends beyond community-acquired to include hospital-acquired infections, many of which are particularly challenging to treat. The presence of resistant strains of microorganisms contributes significantly to the difficulty of managing such infections, ultimately resulting in considerable annual mortality [3]. In the context of microbial infections, the transition of microorganisms from planktonic forms to biofilms represents a critical shift that significantly impacts the effectiveness of antimicrobial treatments [4]. While planktonic forms serve as the starting point for evaluating antimicrobial activity and interactions [5], biofilms present a more complex and resilient state that is highly relevant to the challenges encountered in clinical settings [6]. Understanding the interplay between the planktonic and biofilm states is essential for developing comprehensive treatment strategies that address the complexities of microbial infections in real-life scenarios. The most traditional and widely used approaches for the treatment and/or prevention of infections involve the use of antimicrobial agents [7]. However, when these agents are applied in suboptimal concentrations, they can increase the likelihood of drug-resistant microorganisms emerging [8]. This can lead to future infections that are more challenging to eradicate due to the development of resistance. Consequently, the application of antimicrobial agents alone has limited effectiveness in both treating and preventing infections [9]. In modern practice, novel antimicrobial agents are frequently employed in conjunction with conventional approaches for the treatment and/or prevention of infections. One such category of agents is antimicrobial peptides (AMPs), a subset of host defense peptides (HDPs). HDPs can demonstrate a wide range of actions, and the majority of these actions provide direct effects, such as antimicrobial activity, or indirect effects, such as immunomodulatory/anti-inflammatory defense against pathogens [10]. AMPs can engage with microbial membranes non-specifically due to their amphiphilic nature and positive charge, and AMPs have a low potential to induce drug resistance [11]. Nonetheless, AMPs also have some disadvantages. Maintaining peptide activity and stability under physiological conditions is a critical need for optimum efficacy. The stability is determined by their susceptibility to enzyme degradation and inhibition by proteins, salts, and ions found in the environment. In addition, pathogens may protect themselves from AMPs by producing peptide-degrading enzymes [12].

Both conventional and novel approaches may be found to be insufficient in the treatment and/or prevention of infections [13, 14]. Different approaches are currently in progress to increase antimicrobial effectiveness, and one method is to use two different antimicrobial agents in combination [15]. Combining antimicrobial agents may allow the targeting of various microorganisms, improving treatment and/or prevention efficacy, reducing the effective concentration of antimicrobial agents, and overall reducing the treatment cost [16]. The evaluation of antimicrobial interactions often involves experimental procedures such as the checkerboard assay, which enables the testing of multiple concentrations of each compound to assess their combined effects [17]. It is important to note that while the checkerboard assay serves as the experimental platform for assessing antimicrobial interactions, the FIC index itself is not a direct experimental test but rather a quantitative measure derived from the experimental data [18]. As such, the FIC index plays a crucial role in categorizing the combined effects of antimicrobial agents, offering valuable insights into their synergistic potential. Desired concentrations for the synergistic effect of two different antimicrobial agents may be found as a result of the series of experiments. Still, the experiments are time-consuming, and the costs are undeniable as they will consume a lot of material and resources. On the other hand, there are a lot of studies in the literature in which different biological outcomes are predicted by machine learning (ML) algorithms by transforming the existing data into an artificial intelligence (AI) model without the need for experimental studies [19, 20]. The high accuracy values of ML in the experimental science area [21] are also promising for different fields, especially for AMP studies [22].

The introduction of modern technological advances in AI has altered the prospects of biomedicine. AI is being applied to solve complicated problems in this area [23]. ML is a subfield of AI, and the goal of ML is to create algorithms that guide machines on how to access data and utilize it to learn a given task [24]. ML studies have many different applications in the field of AMPs and are frequently used. The findings obtained from studies encourage the examination of the synergistic effect of AMPs and antimicrobial agents with ML algorithms. Although many ML algorithms predict various functions of AMPs, it was found that there is no ML-based algorithm that predicts the synergistic effects of AMPs and antimicrobial agents. Considering the lack of literature, this study aims to predict the synergistic effect of various antimicrobial agents with different AMPs by predicting the FIC index with several ML methods, and to the best of our knowledge, this is the first study in this specific field. The existence of such a model may save researchers time, effort, and resources in the laboratory for an appropriate combination of antimicrobial agents and AMP.

## Related studies

ML applications are widely used in medicine and biomedical fields for the prediction of desired biological outcomes. For instance, Furxhi et al. [25] presented a neurotoxicity classification model to predict cell viability. They developed a model based on a random forest (RF) algorithm, and the reliability validation test of the model’s performance achieved an accuracy score of 72%. In another study, Shaban et al. [26] introduced an ML-based predictive modeling approach to predict the *in vitro* antibiofilm activity of antibiotics. They developed three models based on logistic regression (LR), decision tree (DT), and RF algorithms with accuracy scores of 67±6.1%, 73±5.8%, and 74±5%, respectively. Besides using ML algorithms to predict a biological outcome, AI applications are also commonly employed in the field of AMPs.

Progress in AMP studies has fueled ongoing efforts to develop computational approaches for accurate AMP prediction to significantly reduce the effort and time required for experimental identification [27]. To date, various computational methods for the assessment, prediction, and design of new AMPs have been developed. AVPpred [28], BIPEP [29], AmPEP [30], ClassAMP [31], and DBAASP [32] are a few examples. Furthermore, computational prediction of the activities of AMPs against pathogens as well as their structural properties provides a supportive technique for the time-consuming and labor-intensive experimental characterization of AMPs by shortlisting potential AMP candidates for later experimental validation [33]. Plisson et al. [19] constructed ML algorithms and outlier detection techniques to guarantee robust predictions for AMP discovery and the design of new peptides with lower hemolytic activity. They evaluated 14 binary classification algorithms, and their best model predicted the hemolytic tendency of any peptide sequence with an accuracy of 95-97%. In another study, Li et al. [20] sought to identify factors regulating selectivity by correlating peptide sequence information with bioactivity data using the RF algorithm. Out-of-bag prediction generated satisfactory predictive models with accuracies over 0.80. Model interpretation using variable significance metrics and partial dependency plots revealed that the distribution patterns and composition of molecular charge and solubility-related factors strongly influenced selectivity. In a different study, Gull et al. [22] developed AMAP, which is an ML-based model to predict the biological activity of peptides with an emphasis on antimicrobial activity predictions. Their findings demonstrated that their models developed with the non-linear support vector machines (SVM) algorithm and the extreme gradient boost (XGBoost) algorithm separately are capable of accurately predicting the biological activity of new peptide sequences with an accuracy score of 97%.

Prediction of various biological outcomes using ML techniques has lately been a popular bioinformatics research topic. AMP studies are among the most studied areas to provide a better understanding of the mechanisms of AMPs as well. Despite numerous AMP properties and biological outcomes that have been investigated using various AI techniques, no research has been reported previously on predicting the synergistic effects of AMPs and other antimicrobial agents. In light of the findings, this paper presents a novel technique to accurately predict the interactions between AMPs and antimicrobial agents in terms of the FIC index using supervised ML algorithms for the first time.

## Results

### Data interpretation

The data related to the AMP features were collected from the DBAASP [32] database by applying the necessary extraction criteria. For the use of synergistic effects, information about antimicrobial agents of interest was obtained from the DrugBank [34] database. Data was collected to use supervised ML algorithms to predict the synergistic effects between AMPs and other antimicrobial agents. After determining the predictors and outcome, rows with missing values were removed. In total, 407 rows of data were collected. These observations from the obtained data were used in the construction, training, validation, and testing of the ML models.

**Fig. 1:**
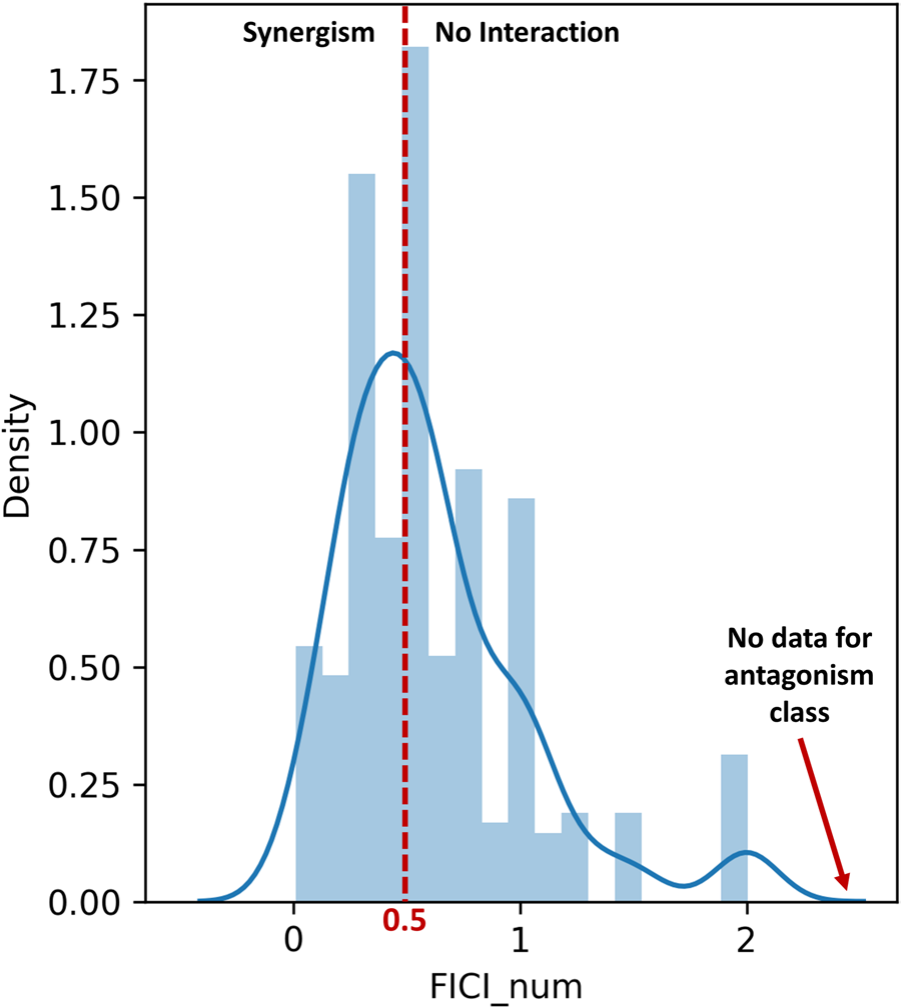
The data distribution of the FIC index (FICI_num: FIC index numeric)

Among predictors, several antimicrobial agents and microbial characteristics were based on nominal data. Table 1 represents the nominal predictors with their labels and frequencies. Among microbial characteristic predictors, the one with the highest observation of a microbial species was the *P. aeruginosa* (32.2%). Other than nominal data, some of the predictors contain numeric data as well. Table 2 represents the descriptive statistics of numeric variables. For minimum inhibitory concentration (MIC), some values were given in $\mu$M, while others were given in $\mu$g/mL. Values given as $\mu$M were converted to $\mu$g/mL for unit integrity. Furthermore, the FIC index was determined as the output. Among the collected FIC data, 199 corresponded to instances of synergistic effects, while 208 were associated with scenarios lacking significant interaction. There are 101 unique values of the FIC index among the 407 observations. While the minimum FIC value was 0.01, the maximum was 1.98, and the mean value of the FIC values was 0.63. Figure 1 presents the FIC index’s data distribution. The graph indicates that the data distribution of the FIC index is most concentrated between 0 and 1. Moreover, the distribution of the data confirms the absence of an antagonistic class in our dataset, where antimicrobial agent combinations exhibit FIC indices exceeding 4.

**Table 1:** Nominal predictors, their categories, and labels with the frequencies of unique values (Frequency presents the percentage of the related label in a total observation of 407)

Category	Variables	Labels (Frequency (%))
**Antimicrobial Agent Characteristics**	**Antimicrobial Agent Name**	Amikacin (2.7), Amoxicillin (1.2), Amphotericin B (1.0), Ampicillin (2.5), Azithromycin (2.5), Bacitracin (0.2), Cefepime (1.0), Cefotaxime (0.7), Ceftazidime (2.7), Ceftriaxone (1.0), Cephalothin (0.7), Chloramphenicol (3.2), Ciprofloxacin (7.6), Clarithromycin (1.5), Clindamycin (1.2), Colistin (0.7), Doripenem (1.0), Doxycycline (0.7), Erythromycin (7.1), Fluconazole (1.2), Gentamicin (9.8), Imipenem (4.2), Kanamycin (1.7), Levofloxacin (0.5), Meropenem (3.2), Minocycline (0.7), Novobiocin (1.0), Ofloxacin (1.0), Oxacillin (1.7), Penicillin (0.5), Piperacillin (1.5), Polymyxin B (8.1), Rifampicin (10.6), Streptomycin (0.7), Tetracycline (2.9), Tobramycin (1.5), Vancomycin (9.8)
	**Antimicrobial Agent Class**	Aminocoumarin (1.0), Aminoglycoside (16.5), Ansamycin (10.6), Azole (1.2), Beta-lactam (21.9), Chloramphenicol (3.2), Fluoroquinolone (9.1), Glycopeptide (9.8), Lincosamide (1.2), Macrolide (12.0), Polymyxin (8.8), Polypeptide (0.2), Tetracycline (4.4)
	**Mechanism of Action**	Antimetabolite (2.0), Cell membrane (11.1), Cell membrane and Nucleic acid (11.3), Cell membrane and Protein (0.7), Cell wall (25.8), Cell wall and Protein (0.7), Nucleic acid (25.1), Nucleic acid and Antimetabolite (0.5), Protein (22.9)
	**Gram Activity**	Gram-negative (11.3), Gram-positive (14.0), Gram-positive and Gram-negative (74.7)
**Microbial Characteristics**	**Microbial Species**	*A. baumannii* (7.9), *C. albicans* (1.2), *C. auris* (0.5), *C. neoformans* (0.5), *E. faecalis* (0.7), *E. coli* (23.3), *K. aerogenes* (0.5), *K. pneumoniae* (11.5), *M. luteus* (0.7), *M. bovis* (0.2), *M. smegmatis* (0.7), *M. tuberculosis* (0.2), *P. aeruginosa* (32.2), *S. aureus* (15.0), *S. epidermidis* (4.2), *S. pyogenes* (0.5)
	**Microorganism or Gram Class**	Fungus (0.5), Gram-negative (75.4), Gram-positive (23.6), Gram-positive and Gram-negative (0.5)

**Table 2:** Descriptive statistics of numeric variables (All variables have a total count of 407, FH indicates the fractional helical content)

Category	Variables	Unit	Unique	Mean	SD	Min	Max
**AMP Characteristics**	**Length**	a.a.	20	14.39	5.8	6	37
	**Molecular Weight**	g/mol	65	1847.29	610.31	1044.23	4003.84
	**Normalized Hydrophobicity**	$\Delta$G	59	0.6	1.1	-1.86	3.23
	**Net Charge**	N/A	11	5.86	2.12	1	11
	**Isoelectric Point**	pH(I)	18	13.63	0.96	9.93	14
	**Penetration Depth**	Å	13	16.26	5.04	10	30
	**Tilt Angle**	°	46	85.89	28.86	13	169
	**Disordered Conformation Propensity**	FH	58	-0.31	0.45	-1.46	0.82
	**Linear Moment**	kgxm/s	34	0.32	0.15	0	0.57
	**Amphiphilicity Index**	N/A	60	2.14	1.24	0	5.3
	**Average Hydrophilicity**	$\Delta$G	48	0.03	0.45	-0.98	2.16
	**Ratio of Hydrophilic Residues/Total**	N/A	32	40.13	11.46	10	90
	**MIC of AMP**	$\mu$g/mL	92	34.34	58.17	0.25	512
**Antimicrobial Agent Characteristics**	**Physiological Charge**	N/A	8	1.3	2.14	-2	5
	**LogP**	N/A	36	-1.21	3.38	-8.6	4.1
	**Water Solubility**	mg/mL	36	5.79	15.54	0.01	92.3
	**pKa**	mol/L	35	7.48	4.04	2.42	12.68
	**Molecular Weight**	g/mol	37	677.04	360.61	299.35	1449.3
	**MIC of Antimicrobial Agent**	$\mu$g/mL	167	66.21	161.18	0.001	2048
**Outcome**	**FIC Index**	N/A	101	0.63	0.47	0.01	1.98

### Correlation analysis and feature selection

**Fig. 2:**
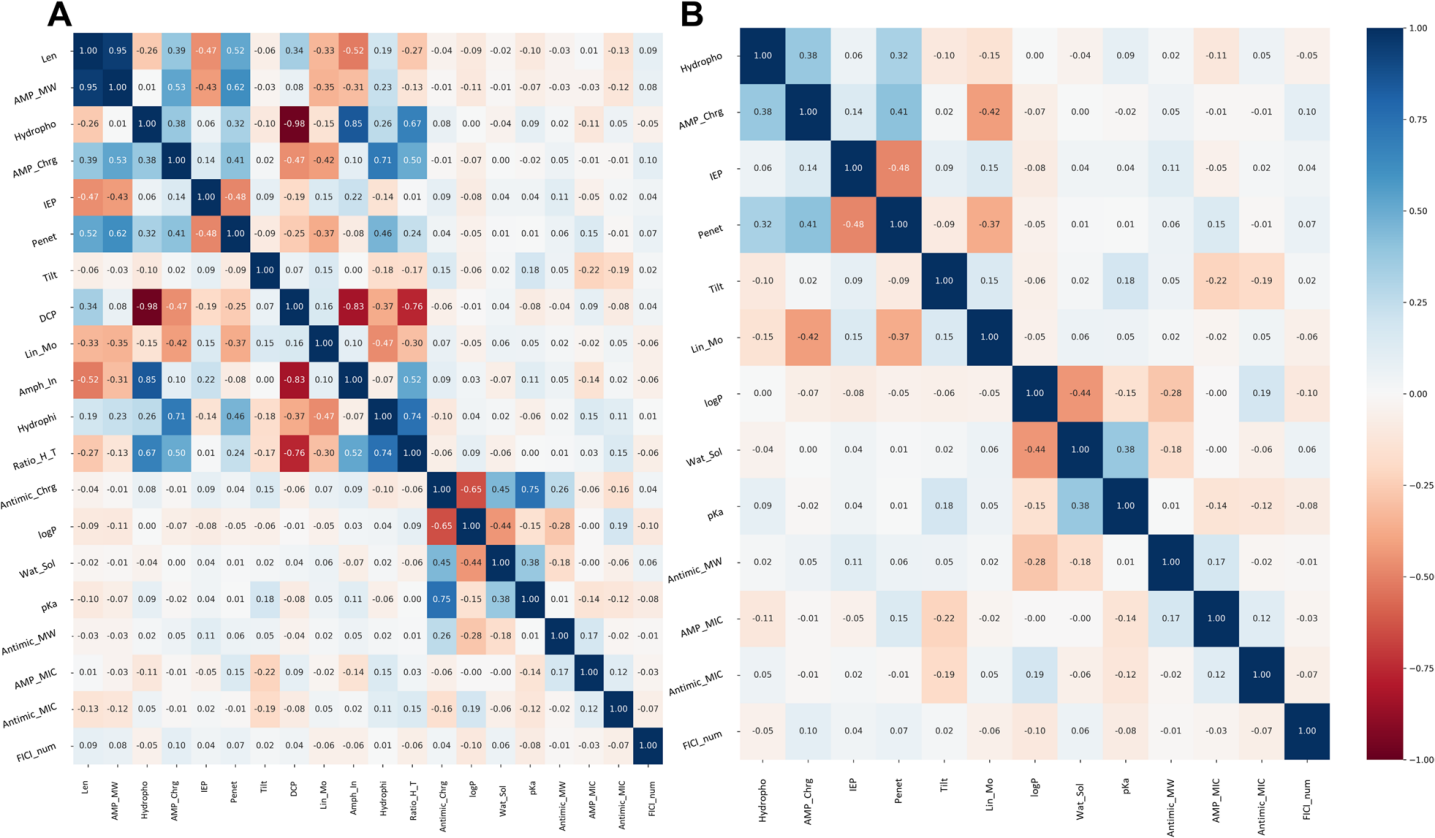
Correlation matrix of predictors and outcome: **(A)** all features and **(B)** after eliminating the correlated features (Syn_Spe: Microbial species in which the synergistic effect of antimicrobials and AMP was investigated, AMP_MIC: MIC of AMP, Antimic_MIC: MIC of antimicrobial agent, Antimic: Antimicrobial agent name, Clss: Antimicrobial class, logP: LogP value of antimicrobial agent, Amph_In: Amphiphilicity index of AMP, Hydrophi: Average hydrophilicity of AMP, Hydropho: Normalized hydrophobicity of AMP, Lin_Mo: Linear moment of AMP, Tilt: Tilt angle of AMP, DCP: Disordered conformation propensity of AMP, AMP_MW: Molecular weight of AMP, Antimic_MW: Molecular weight of antimicrobial agent, MOA: Mechanism of action of antimicrobial, Ratio_H_T: Ratio of hydrophilic residues/total for AMP, pKa: pKa value of antimicrobial, Wat_Sol: Water solubility of antimicrobial agent, Len: Length of AMP, AMP_Chrg: Net charge of AMP, Penet: Penetration depth of AMP, Gram: Microorganism or gram class, Antimic_Chrg: Physiological charge of antimicrobial, Gram_Actv: Gram activity, IEP: Isoelectric point of AMP)

A correlation matrix is a table that illustrates the correlation coefficients for variables. It is a strong tool for summarizing large datasets as well as identifying and visualizing patterns in the data [35]. Coefficients range between -1 and 1, where -1 represents a negative linear correlation, 0 represents that there is no linear correlation, and 1 represents a positive linear correlation. To ascertain how closely related different numerical predictors are to one another, a correlation matrix was developed and presented in Fig. 2A. The model was first built with all the features (*Case 1*). However, several features were correlated with each other. Predictors reported in the literature to have minimal impact on the synergistic effect or antimicrobial activity were eliminated by considering the correlation matrix results. The structure of AMPs is related to a large number of hydrophobic residues and a net positive charge because of the presence of numerous cationic residues such as arginine and lysine, which enable them to fold into amphipathic forms [36]. AMPs can engage with microbial membranes non-specifically due to their amphiphilic nature and positive charge [11]. There were strong correlations between the amphiphilicity index and normalized hydrophobicity (0.85) and between average hydrophilicity and the net charge of AMP (0.71). Based on this information, the amphiphilicity index and average hydrophilicity features were eliminated because hydrophobicity and net charge are more important than other predictors. Also, a strong correlation was observed between the molecular weight and length of AMP (0.95), as expected. For that reason, the length of the AMP predictor was eliminated. Furthermore, a high correlation was observed between the disordered conformation propensity and the normalized hydrophobicity of AMP (-0.98). However, as mentioned before, normalized hydrophobicity has a greater impact on the synergistic effect. That is why the disordered conformation propensity feature was also eliminated. In a study, it was observed that reducing the positive charge of the antimicrobial agent did not change the antimicrobial activity, but decreasing the lipophilicity decreased the activity [37]. Based on this study, it was concluded that lipophilicity, that is, the logP value, is more important than the charge of the antimicrobial agent in terms of activity. Therefore, the charge for the antimicrobial agent has also been eliminated. Moreover, when the charge of the antimicrobial agent was eliminated, its strong correlation with the pKa value (0.75) was also eliminated. One of the two features with a correlation coefficient greater than 0.5 or smaller than -0.5 was removed, and the model was rebuilt after removing correlated features (*Case 2*). Figure 2B represents the correlation matrix after removing the highly correlated predictors. The graph indicates that there is no predictor with a high correlation.

### Effect of preprocessing

**Fig. 3:**
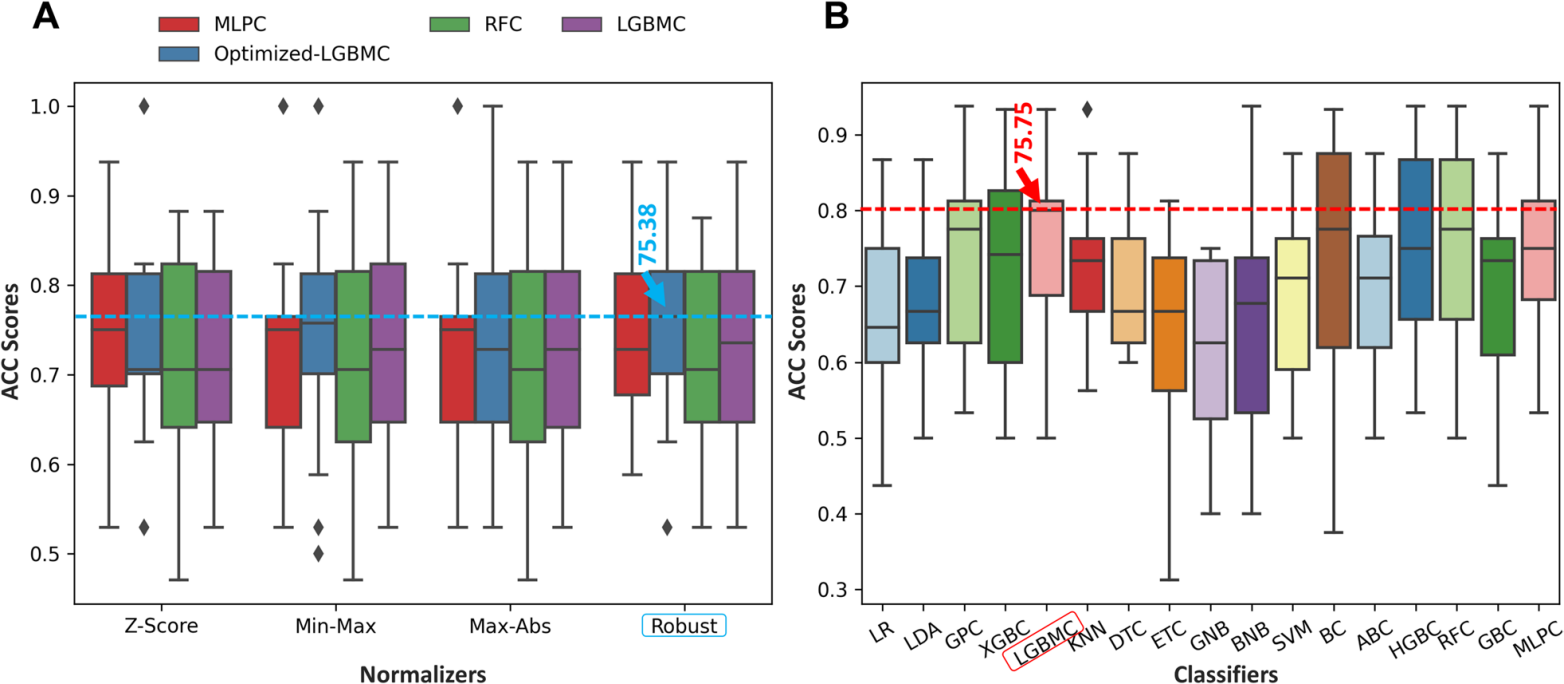
A box-plot representation of accuracy scores of **(A)** different normalization methods and **(B)** different classifiers (The horizontal lines in the boxes indicate the median value of ACCs)

After the obtained observations were evaluated, the data were preprocessed before proceeding to the model development stage. Due to the imbalance in observations among classes, the Synthetic Minority Oversampling Technique (SMOTE) method was used to generate 9 new observations, and the total number of observations for both groups was equalized to 208. Furthermore, four different normalization methods were evaluated in the scope of this study. Their accuracy values were compared with different ML algorithms, and the most accurate normalization method was implemented in the remaining phases of the study. Figure 3A represents the accuracy values of different normalization methods over a variety of ML algorithms. In order to compare the accuracy values of different normalization methods, Multilayer Perceptron Classifiers (MLPC), Random Forest Classifier (RFC), Light Gradient Boosted Machine Classifier (LGBMC), and optimized LGBMC (oLGBMC) classifiers were utilized. oLGMBC is a hyperparameter-optimized version of the LGBMC classifier using the *tpot* classifier. The accuracy values after the robust normalization method for the MLPC, oLGBMC, RFC, and LGBMC classifiers were 73.92%, 75.38%, 72.08%, and 73.93%, respectively. The accuracy value of the oLGMBC model before normalization was 70.53%, whereas after normalization this score increased to 75.38%, demonstrating the impact of the normalization method on the classification performance of the model. A robust normalization technique was selected as the best normalization technique and was adapted before model development. After rescaling the numeric predictors, nominal data were converted to numerical data using the one-hot encoding (OHE) method. Then, the data number of the different classes was equalized to 208 with the resampling method. A one-way ANOVA test was conducted to determine whether a resampling method has statistical significance on the model’s performance, and the *p*-value cutoff was established as .005. After ANOVA, $p>.995$ was obtained, and it can be concluded that the effect of the resampling method on classification performance is not statistically significant. After preprocessing, all the data is ready to train the model. A 3-repeated 5-fold cross-validation method was utilized for the validation of the model. In this study, the *tpot* classifier was employed for the optimization of the hyperparameters. For the genetic algorithm-based *tpot* classifier, the generation size was set to 150, the population size to 100, and the offspring size to 25. Informed searches were conducted with a traditional cross-validation strategy to determine the best pipeline. An Intel^®^ CoreTM i9-10940X CPU and 64 GB RAM were used to develop all supervised ML models. The accuracy of the oLGMBC model increased from 72.43% to 75.38% after hyperparameter tuning.

### Training results

Seventeen different classifiers were adapted for this study, and the accuracy scores of the models developed with these classifiers were evaluated. Figure 3B represents the graph of the validation accuracy scores of 17 different classifiers. The classifier with the highest accuracy value was the LGBMC, with an average validation accuracy of 75.75%. The second and third most accurate models were MLPC and RFC, with average accuracy scores of 75.25% and 75.00%. The model with the lowest accuracy was the Gaussian Naïve Bayes (GNB), with a 61.80% accuracy score. Other models that had lower accuracy values among others were the Extra Tree Classifier (ETC) and the Bernoulli Naïve Bayes (BNB), with accuracy values of 63.80% and 65.90%. There was a 13.95% difference between the accuracy scores of the most accurate model (LGBMC) and the least accurate model (GNB). Although the average accuracy values of the 3 most accurate models were close to each other, the model with the highest accuracy score was the LGBMC; therefore, the LGBMC was adapted for the hyperparameter tuning and the model’s performance evaluation. Table 3 represents the average performance metrics of the 3-repeated 5-fold cross-validation strategy for different classifiers. LGBMC, the best-performing classifier, had accuracy (ACC) and area under the curve (AUC) values for the training of 99.75% and 1.00, and 75.75% and 0.82 for the validation. Moreover, among all classifiers, the F1-score (F1), recall (REC), and precision (PRE) performance metrics had the highest values, reaching 75.30%, 75.65%, and 77.40%, respectively. Other classifiers with a lower elapsed time (ET) were also provided, but their classification accuracy remained low; therefore, they were not adapted for the scope of this study. Furthermore, it was established that LGBMC’s ET was 1.68, which was relatively low in comparison with the average of other classifiers. After the empirical accuracy comparison, a statistical test was also conducted to choose the best classifier. In light of the findings of the statistical analysis, it was determined that the accuracy value of six different classifiers was statistically significant ($p<.005$) when compared to others. Following the model with the highest classification performance (LGBMC), classifiers such as Gaussian Process Classifier (GPC), Bagging classifiers (BC), Histogram Gradient Boosting Classifier (HGBC), RFC, and MLPC have also been statistically significant ($p<.005$) in terms of their validation accuracy values. However, the LGBMC architecture led to the best average validation accuracy among the others.

**Table 3:** Average performance metrics of the 3-repeated 5-fold cross-validation strategy for different classifiers (ACC, F1, REC, and PRE values are given as percentages (%) and the ET unit is s.)

Classifier	Train		Validation						
	ACC	AUC	ACC	AUC	F1	REC	PRE	ET	*p*-value
LR	77.60	0.85	66.60	0.71	66.20	66.55	67.40	0.76	.803
LDA	80.25	0.88	68.25	0.73	68.00	68.25	69.20	1.56	.544
GPC	99.00	1.00	73.95	0.80	73.75	73.90	74.75	3.68	$<.005$
XGBC	100.00	1.00	72.80	0.81	72.40	72.85	74.10	3.95	.017
**LGBMC**	99.75	1.00	**75.75**	0.82	75.30	75.65	77.40	1.68	$<.005$
KNN	81.95	0.90	72.85	0.77	72.50	72.75	74.05	1.05	.017
DTC	100.00	1.00	70.45	0.70	70.25	70.45	70.95	0.69	.237
ETC	100.00	1.00	63.80	0.64	63.40	63.80	64.55	0.66	.972
GNB	70.20	0.81	61.80	0.71	60.75	61.85	62.75	0.69	$>.999$
BNB	71.45	0.77	65.90	0.70	65.15	65.80	66.35	0.70	.868
SVM	74.65	0.81	68.85	0.72	67.95	68.75	70.35	1.73	.447
BC	97.90	1.00	74.15	0.80	73.80	74.30	75.25	1.42	$<.005$
ABC	85.65	0.94	70.15	0.78	69.15	70.20	72.10	3.63	.252
HGBC	99.80	1.00	74.70	0.82	74.30	74.70	76.20	13.49	$<.005$
RFC	100.00	1.00	75.00	0.82	74.65	74.95	75.95	5.77	$<.005$
GBC	96.95	0.99	69.55	0.81	68.90	69.60	70.75	3.39	.333
MLPC	97.25	1.00	75.25	0.77	75.00	75.30	76.70	28.44	$<.005$

**Fig. 4:**
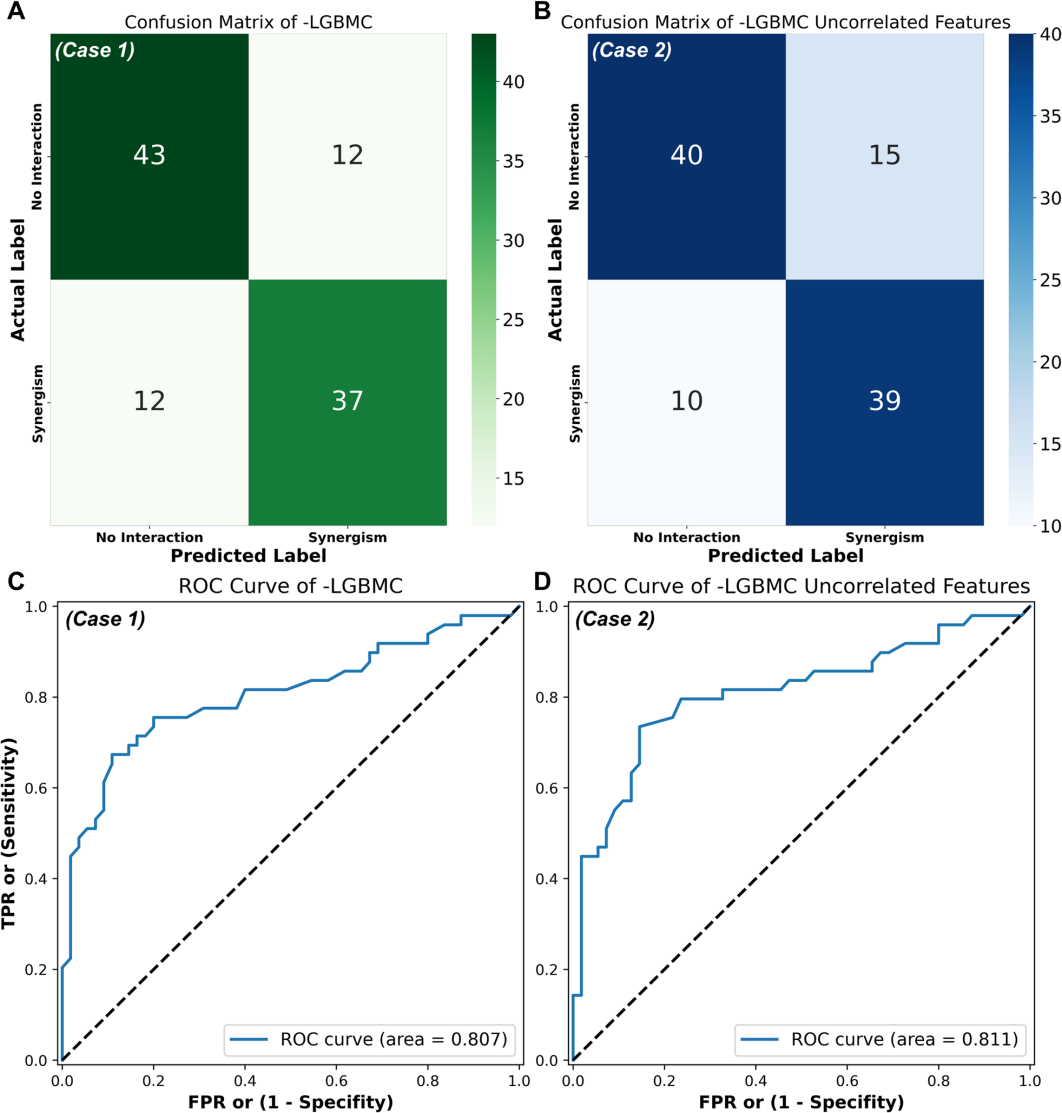
Results of the test phase (model predict) of the eventual oLGBMC model: **(A)** confusion matrix obtained by using whole feature set (*Case 1*), **(B)** confusion matrix after eliminating the correlated features (*Case 2*), **(C)** ROC curve obtained by using whole feature set (*Case 1*), and **(D)** ROC curve after eliminating the correlated features (*Case 2*)

After the comprehensive comparison of classifiers, normalization techniques, and feature analysis, an optimized version of LGBMC (oLGBMC) was determined for the eventual classifier with robust normalization. Then, the classification models were adopted using oLGBMC for the rest of this study. In the test phase, the oLGBMC model achieved 76.92% ACC and 80.71% AUC. Furthermore, F1, REC, and PRE values were yielded as 78.18%. Figure 4A represents the confusion matrix obtained in the test phase of oLGBMC (*Case 1*). There were 55 data points with an FIC index greater than 0.5 (No Interaction). The model predicted 43 of them correctly (true negative) and 12 of them incorrectly (false negative). Also, there were 49 data points with an FIC index less than or equal to 0.5 (Synergism). The model predicted 37 of them correctly (true positive) and 12 of them incorrectly (false positive). If expressed as a percentage, the model correctly predicted 78.2% of the data with no interaction and correctly predicted 75.5% of the data with synergism. The receiver operating characteristic (ROC) curve is a graph that depicts model performance at all classification thresholds. This curve depicts two parameters: True Positive Rate (TPR), and False Positive Rate (FPR). Moreover, AUC measures the whole two-dimensional area under the ROC curve, and these values vary from 0 to 1 [38]. The AUC value of the model was 0.807 (see Fig. 4C for *Case 1*). Figure 4B represents the confusion matrix obtained by the supervised ML model generated by eliminating the correlated features (*Case 2*). The results indicate that the model prediction performance for the “No Interaction” group decreased from 78.2% to 72.7% when compared with the confusion matrix including all the features, while the prediction accuracy for the “Synergism” group increased from 75.5% to 79.6%. The ROC curve generated after the correlated features were eliminated (*Case 2*) is presented in Fig. 4D. The achieved AUC value was 0.811. The results demonstrated that removing the correlated features for predicting the FIC index not only increased the AUC value and the model performance but also significantly reduced the computational cost compared to the case where all features were employed to build the model.

### Contribution of features

**Fig. 5:**
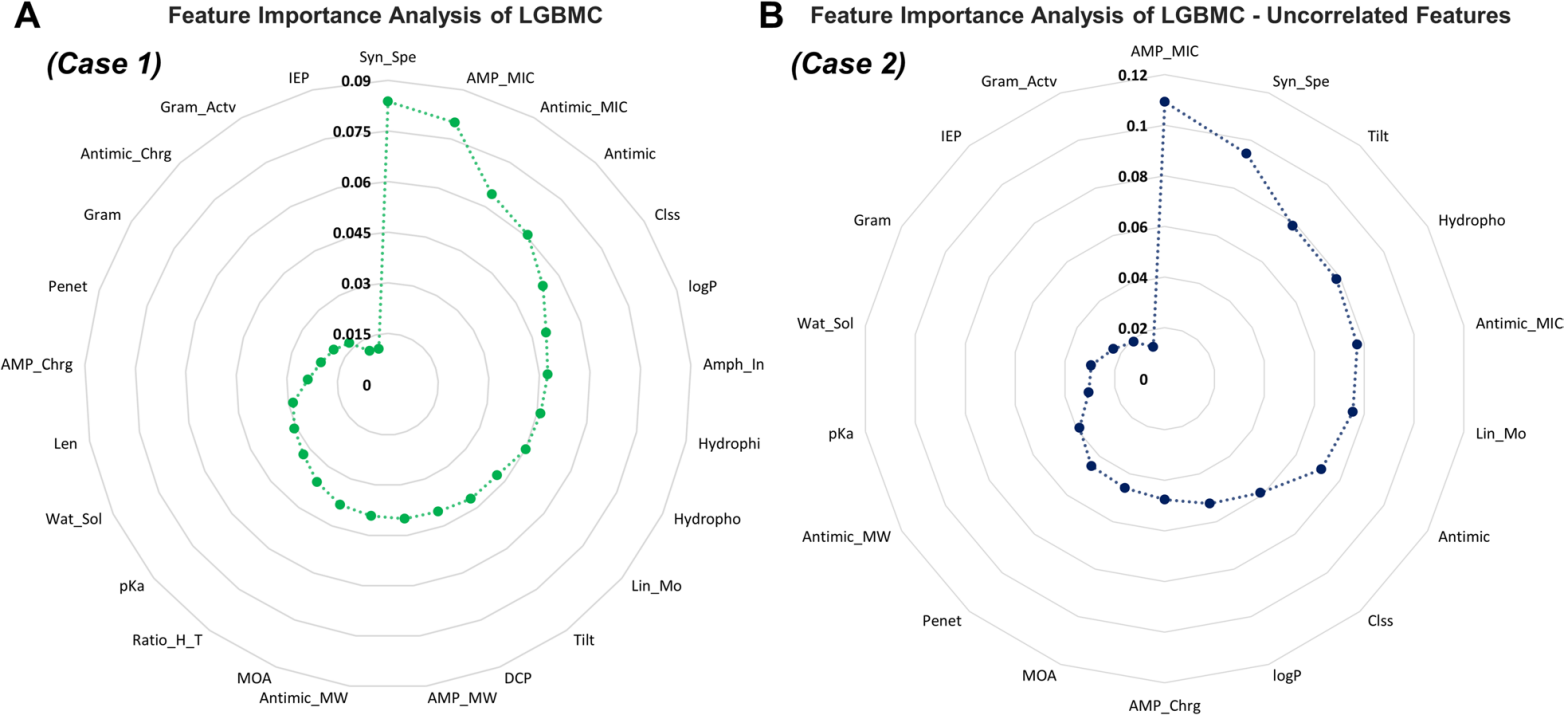
Feature importance analysis results for oLGBMC model: **(A)** by using whole feature set (*Case 1*) and **(B)** after eliminating the correlated features (*Case 2*) (Syn_Spe: Microbial species in which the synergistic effect of antimicrobials and AMP was investigated, AMP_MIC: MIC of AMP, Antimic_MIC: MIC of antimicrobial agent, Antimic: Antimicrobial agent name, Clss: Antimicrobial class, logP: LogP value of antimicrobial agent, Amph_In: Amphiphilicity index of AMP, Hydrophi: Average hydrophilicity of AMP, Hydropho: Normalized hydrophobicity of AMP, Lin_Mo: Linear moment of AMP, Tilt: Tilt angle of AMP, DCP: Disordered conformation propensity of AMP, AMP_MW: Molecular weight of AMP, Antimic_MW: Molecular weight of antimicrobial, MOA: Mechanism of action of antimicrobial, Ratio_H_T: Ratio of hydrophilic residues/total for AMP, pKa: pKa value of antimicrobial, Wat_Sol: Water solubility of antimicrobial agent, Len: Length of AMP, AMP_Chrg: Net charge of AMP, Penet: Penetration depth of AMP, Gram: Microorganism or gram class, Antimic_Chrg: Physiological charge of antimicrobial, Gram_Actv: Gram activity, IEP: Isoelectric point of AMP)

The effect of the importance of predictors on the performance of the model was measured in terms of information gain. Feature importance analyses of the models that were built with all predictors (*Case 1*) and after removing the correlated features (*Case 2*) are represented in Fig. 5A and B. It was concluded that the most important feature among all features was the microorganism species in which the synergistic effect of antimicrobial agents and AMPs was investigated (see Fig. 5A). The second and third most important features were the MIC values of the AMPs and antimicrobial agents when used alone. It was also observed that the least important features were the isoelectric point (IEP) of the AMP, the charge of the antimicrobial agent, and the gram class of the pathogen on which the antimicrobial agent was active. The total feature importance distribution was found as 50%, 40%, and 10% for AMPs, antimicrobial agents, and common features in *Case 1*, while it was 46%, 42%, and 12%, in *Case 2*. Especially in *Case 2*, 7 features were represented for AMP characteristics, with an average of 6.5% importance per feature, while this ratio was represented by 4.6% by 9 features of antimicrobial agents. Therefore, it was established that the AMP characteristics may be more important than the features of the antimicrobial agents in terms of synergistic effect, according to both cases. Besides, the MIC value of the AMP was the most weighted feature overall, according to *Case 2* (Fig. 5B). The microorganism species in which the synergistic effect of antimicrobial agents and AMP was studied was the second-most important feature. These two predictors continue to dominate the model’s performance in terms of weighted feature importance, just as in the first model. Moreover, the tilt angle has ascended to the third-most significant feature after the removal of the correlated predictors. It is evident from the graphs that the feature importance analysis results have altered as a result of the model’s reconstruction and the removal of several predictors. For the least important features, there have been no significant changes after rebuilding the model.

### External testing and model generalizability

**Fig. 6:**
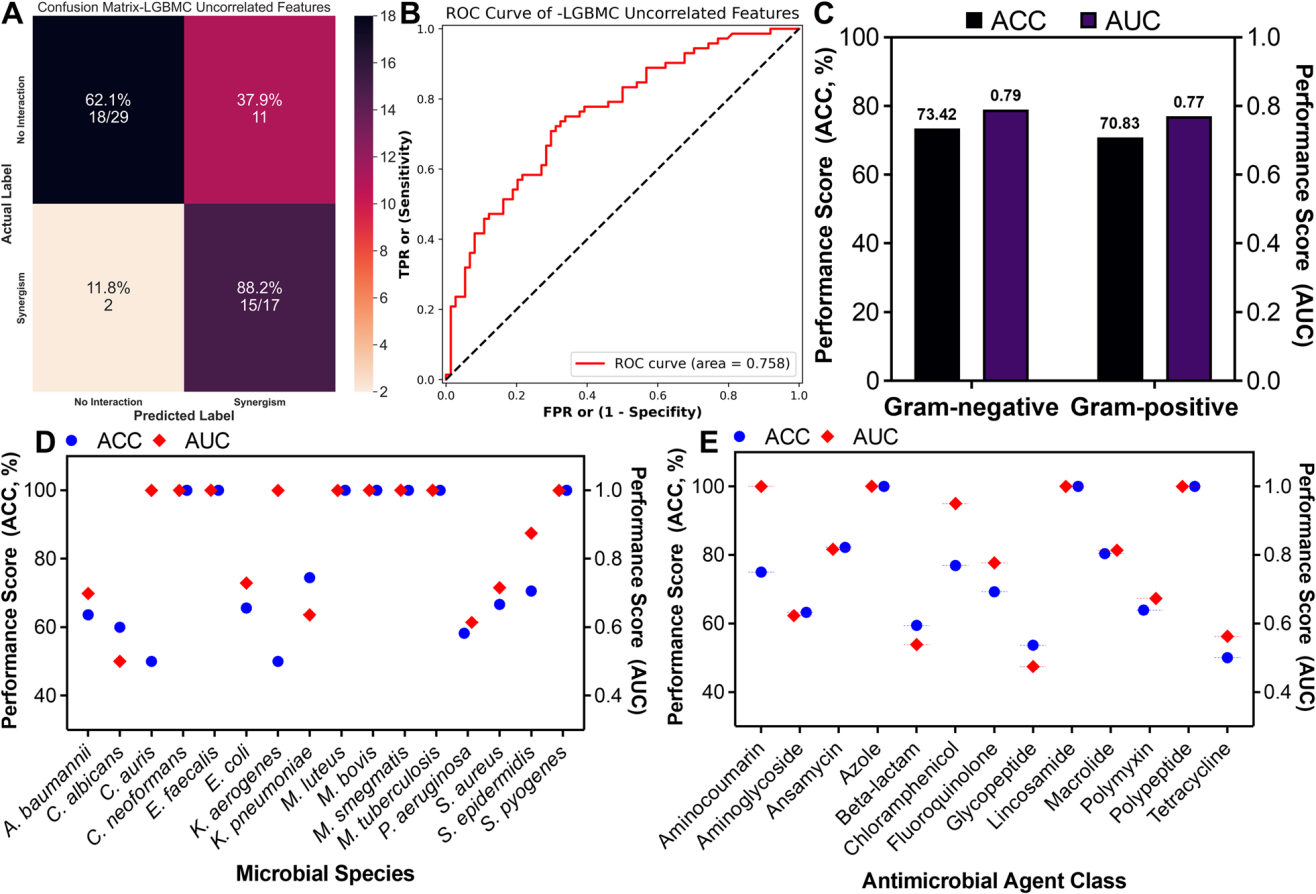
External test set and leave-one-out strategy results for the model generalizability. **A** Confusion matrix of the trained model on external test data, **(B)** ROC curve for the external test dataset. **C** performance scores for Gram-positive and Gram-negative categorization of external tests including ACC and AUC. ACC and AUC distributions across micro-organism and -agent: **(D)** leave-one-microorganism-out approach and **(E)** leave-one-microbial agent-out approach

To further assess the performance of the trained model, external tests were conducted employing recently acquired data, in line with the absence of antagonism classes observed in the original dataset. The performance metrics from this evaluation revealed an accuracy score of 71.74% (Fig. 6A) and an AUC score of 0.76 (Fig. 6B). For further evaluation of the model’s generalizability, the newly collected external test data was categorized into two groups: Gram-negative and Gram-positive microorganisms. Despite the initial dataset’s skewed distribution in favor of Gram-negative species (comprising 75.4% of the data), the model exhibited consistent performance across both categories. Specifically, the Gram-negative class had an accuracy value of 73.42%, while the Gram-positive class achieved an accuracy of 70.73% (Fig. 6C). Moreover, separate evaluations based on the leave-one-out strategy for each unique microbial species, as well as individual antimicrobial agents, were conducted to provide a more detailed assessment of the model’s performance and generalizability. These evaluations yielded average accuracy values of 78.70% for microbial species (Fig. 6D) and 72.86% for antimicrobial agents (Fig. 6E), confirming the model’s reliability and effectiveness in diverse scenarios.

## Discussion

### Significance of ML implementation in AMP research

This study presents an implementation of ML in the field of AMP, apart from existing literature, to predict the synergistic effect with antimicrobial agents. Determining the synergistic effect between AMPs and antimicrobial agents as combination therapy is challenging. This is because there is already a wide variety of AMPs and antimicrobial agents available on the market, making it necessary to conduct time- and resource-consuming laboratory experiments to determine the synergistic effects of various combinations of all. In the literature, there are numerous AI applications with diverse characteristics for AMPs to predict features of interest before conducting any laboratory experiments. However, despite the number of studies, there is no reported study predicting the synergistic effect of AMPs with other antimicrobial agents using the FIC index. The existence of numerous AI studies in the realm of AMPs, the growing usage of AI in medicine and biomedical fields, and its biological applications are all encouraging for the proposed study. Such a study will provide insight into the synergistic effect of AMPs and other antimicrobial agents in combination, provide automatic prediction without the need for long and expensive laboratory experiments, and also shed light on the identification of the most important features that cause the synergistic effect.

### Impact of preprocessing and model selection

In the scope of this study, multiple preprocessing steps along with different classification models were implemented since the best combination was selected to build and train the eventual ML model. Moreover, the completed training, validation, and testing outcomes were fairly and comprehensively assessed. This led to the implementation of several classification scenarios, parameter tuning methods, cross-validation techniques, statistical analyses, and the presentation of robust statistical measures. Models developed using raw data before normalization techniques had lower prediction accuracy performances, and the obtained dataset’s broad data ranges for features are the primary cause, as stated in the literature [39]. Before training the model, the dataset ranges for each feature were restricted by normalization techniques to simplify the training process. After normalization, the model’s prediction performance increased, and the robust scaling method demonstrated the highest accuracy. It can be deduced that the normalization method has a great impact on model accuracy, and after the normalization step, different ML classification models were generated to predict synergistic effects with acceptable accuracy values.

### Assessment of LGBMC model performance and effect of feature elimination on model accuracy

LGBMC is a type of DT-based gradient boosting framework that can be employed for classification and many other ML applications. Gradient-boosted DTs continuously train an ensemble of superficial DTs, with each cycle using the error residuals from the previous model to fit the next model. By boosting, bias and underfitting can be lowered [40]. LGBMC has higher accuracy than any other boosting algorithm, and it is compatible with large datasets. In line with these advantages as discussed in the literature [41], it has been revealed that the optimized version of LGBMC (oLGBMC) was the best model with the highest test accuracy of 76.92% in the scope of this study. After obtaining the most accurate model, parameter tuning was performed both to validate the performance and to achieve higher accuracy scores by optimizing the parameters within the model. The achieved results underscore that parameter tuning significantly influences model accuracy, with the observed enhancement in accuracy due to parameter tuning being widely acknowledged in the existing literature [42]. Moreover, the oLGBMC model’s classification performance was proven by this situation once again. Performance metrics were evaluated for oLGBMC to discuss the classification results and prediction performance of the model in more detail. All performance metrics outperformed the oLGBMC model when compared to other classifiers. Furthermore, it can be deduced that the oLGBMC model can predict the “No interaction” class with higher accuracy than the “Synergism” class, which might assist in excluding particular antimicrobial agents for expected synergism when used in combination with AMPs. Considering all the results, the prediction accuracy of the oLGBMC is relatively lower when compared with the AI studies conducted in the field of AMPs; however, when considering the studies in the literature that predict a specific experimental biological output, it can be concluded that the classification accuracy of the proposed study is promising. Furthermore, this study is the first in the field of AMPs to predict the synergistic effects of two different agents in terms of the FIC index and may provide a basis in this field.

The classification performance of the model was evaluated both in the case of using all the features and in comparison with the model created after eliminating the correlated features. If those two cases are compared, although there is a negligible decrease in the accuracy value (from 76.92% to 75.96% in terms of test accuracies) after eliminating the correlated features, the computational cost and the model’s complexity have decreased significantly. Thus, the accuracy decreased by only 0.96% while 7 uncorrelated features were eliminated. It can be concluded that the model can successfully predict the synergistic effect of AMPs and other antimicrobial agents without the inclusion of correlated features (see Figs. 2 and 4). This shows that the model developed with uncorrelated features (*Case 2*) may allow better associations between the outcome and uncorrelated predictors by increasing the effectiveness of those features used in the training phase.

### Insights from feature importance analysis

Feature importance analysis was conducted to determine the features that have the most impact on the model’s performance and to compare these features in two different trained models (two different cases) with the literature findings. In the literature, it was revealed that the amino acid residues, net charge, amphipathicity, hydrophobicity, and structural characteristics of AMPs were shown to be the most critical physicochemical and structural criteria for their antimicrobial action [43]. Each of these parameters is related to each other, and an alteration in one would cause changes in others. Changes in sequence, length, and charge will alter the hydrophobicity of the AMPs, which has a direct impact on their antimicrobial activity [44]. The relation between activity and charge is not straightforward, as there are various cases of direct, indirect, or indeed inverse correlations between the two. An increase in the positive charge enhances their antimicrobial activity; however, there is a point after which the activity is no longer enhanced. Moreover, excessive positive net charge results in lower antimicrobial activity [45]. Also, according to several studies, increasing hydrophobicity has been linked to increased activity within a particular range [46]. However, when a certain threshold is crossed, an AMP’s hemolytic activity increases considerably while its cell selectivity reduces, and therefore activity decreases [44]. Similarly, the antimicrobial activities of other antimicrobial agents vary according to their characteristic features [47]. The main point here is that when these two different antimicrobial agents are used together, how their individual characteristic features contribute to the synergistic effect. For instance, it was expected that both MIC values of the agents were of great importance since the FIC index value is inversely proportional to the MIC values of the agents when they are used alone [48]. While this situation is provided for the model trained with all features (*Case 1*), it is different than expected in the model trained after the elimination of the correlated features (see Fig. 5 for *Case 2*). The MIC of AMPs and microbial species are the most weighted features for feature importance analysis in both cases. This is an expected output for the MIC of the AMP, which is directly correlated to the FIC index. Furthermore, it is not surprising that microbial species are among the most important features because two antimicrobial agents have a synergistic effect on them. When the least important features were examined, it was concluded that the IEP of the AMP and the gram class of the pathogen were common features for both models. In [49], it was demonstrated that AMPs and non-AMPs have similar average IEPs. Based on this, it can be deduced that the antimicrobial activities of AMPs may not be dependent on the IEP, and it can be further concluded that this is why it was among the least important features in predicting the FIC index. Hence, the findings in the literature for the antimicrobial effect of AMPs and antimicrobial agents are in line with the outputs of the model’s feature importance analysis.

### Considerations for model generalizability

The results from the external test (Fig. 6) affirm the model’s ability to maintain its predictive accuracy when confronted with new datasets, underscoring its resilience and adaptability to novel data sources. The achieved levels of ACC and AUC serve as reliable indicators of the model’s capability in predicting antimicrobial interactions, even when faced with data that wasn’t part of its initial training. Moreover, the minor 3% variation in accuracy between the Gram-negative and Gram-positive classes in the external test highlights the model’s effectiveness in generalizing its predictions, regardless of data imbalances. This adaptability extends to accommodating diverse microbial classifications and providing precise predictions across varying data distributions. The model’s performance scores for individual microbial species and antimicrobial agents further showcase its potential utility in tailored applications. Additionally, the leave-one-out approach for microbial species and antimicrobial agents yielded promising accuracy values, reinforcing the model’s robustness and real-world applicability. Our comprehensive evaluation, encompassing the model’s performance on new data, its robustness across gram classes, and its accuracy scores for unique microbial species and antimicrobial agents, offers valuable insights into the practical implications of our predictive model. Beyond its high accuracy in predicting antimicrobial synergy, the model demonstrates exceptional adaptability and versatility. This adaptability is particularly significant in clinical and research contexts, where microbial strains and antimicrobial agents exhibit wide variability. The model’s consistent performance across diverse microbial classes, data distributions, and individual combinations positions it as a valuable tool for guiding combinational approach strategies.

### Limitations and future directions

In addition to the promising results of this study, some limitations need to be acknowledged. Firstly, while our dataset has yielded promising results, it is important to note that it does not contain an antagonism class, which corresponds to FIC index values greater than 4. This absence may limit the comprehensiveness of our study, as it does not encompass the full spectrum of potential interactions between antimicrobial agents and antimicrobial peptides. Future studies should aim to collect data encompassing all classes, including antagonistic interactions, to provide a more comprehensive understanding of FIC index prediction performance by ML models. Furthermore, a limitation of our current dataset lies in its size. While our model has demonstrated promising performance, a larger dataset with a low similarity rate is necessary to further enhance the model’s validation. Larger datasets not only contribute to the robustness of the model but also help reduce variance. Additionally, it’s worth noting that our model’s performance was confined to the data obtained from the databases we utilized, and achieving a perfect balance for all predictors can be challenging. We acknowledge that the distribution of predictors may not be perfectly balanced due to the dataset’s nature. While this distribution may present a limitation, it is important to emphasize that this aspect is inherent to the dataset itself. While the developed model can assess new data with promising performance, it remains essential to explore the model’s performance on diverse datasets from various sources with a more balanced data distribution. Another limitation of the study lies in its focus on the planktonic form of microorganisms, which may not fully represent the complexities associated with biofilm-related infections. While our research emphasizes the significance of the FIC index in predicting synergistic effects, it is important to recognize that the data obtained from planktonic cultures may not directly correlate with the challenges encountered in biofilm-related infections. The enhanced resilience of biofilms to antimicrobial treatments underscores the need for future studies to incorporate biofilm-related data to bridge the gap between experimental findings and clinical applications. In summary, while our study lays a strong foundation for FIC index prediction using ML models, future research should focus on expanding the dataset to include all classes as well as delving into diverse categorizations of the FIC index via separate ML models. Additionally, considering the different states of microorganisms for ML studies, such as biofilm-related infections, would significantly enhance the clinical relevance of the findings. These endeavors will contribute to a more comprehensive and robust understanding of antimicrobial interactions.

At last, AI is beneficial in a variety of medical and biomedical areas, which motivates its use in combinational approaches. This is the first study to demonstrate how ML modeling is capable of quantitatively predicting the synergistic effects of two different antimicrobial agents in the treatment and/or prevention of infections. This study is an innovative study for the future progress of qualitative as well as quantitative prediction models in combinational approaches using ML. The final findings of the study are encouraging, and the model can predict the FIC index automatically without any experimental procedures. The processing of varying distributions of input data by ML has also shown exceptional performance in terms of cost of computation and prediction ability. To conclude, an ML model capable of automatically predicting the FIC index with relatively high accuracy has been developed. The obtained results suggest the possibility of combining ML approaches with combinatorial antimicrobial agent applications to determine the “FIC index”.

## Conclusion

AMPs are getting significant attention from both research and industry, together with clinical interest. With increasing resistance to traditional approaches, the need for novel and effective solutions has become critical. In this study, we attempted to build an advanced ML-based computational technique for identifying the synergistic effects among various AMPs and antimicrobial agents for their further use as combinational approaches for microbial infections. To the best of our knowledge, this study is the first to use ML approaches to investigate the synergistic effect and predict the FIC index. Findings indicated that the developed oLGBMC model outperformed other algorithms, and it can predict the FIC index with a test accuracy of 76.92%. Furthermore, the results of the feature importance analyses were consistent with the literature. In line with the satisfactory results, it is anticipated that this study may shed light on future studies based on a further understanding of whether antimicrobial agents will work synergistically with AMPs and thus may prevent the loss of time and resources spent in trials and laboratory experiments. At last, while there has been a steady improvement in the reported accuracy of AMPs throughout the last decade, there is still room for improvement.

## Methods

**Fig. 7:**
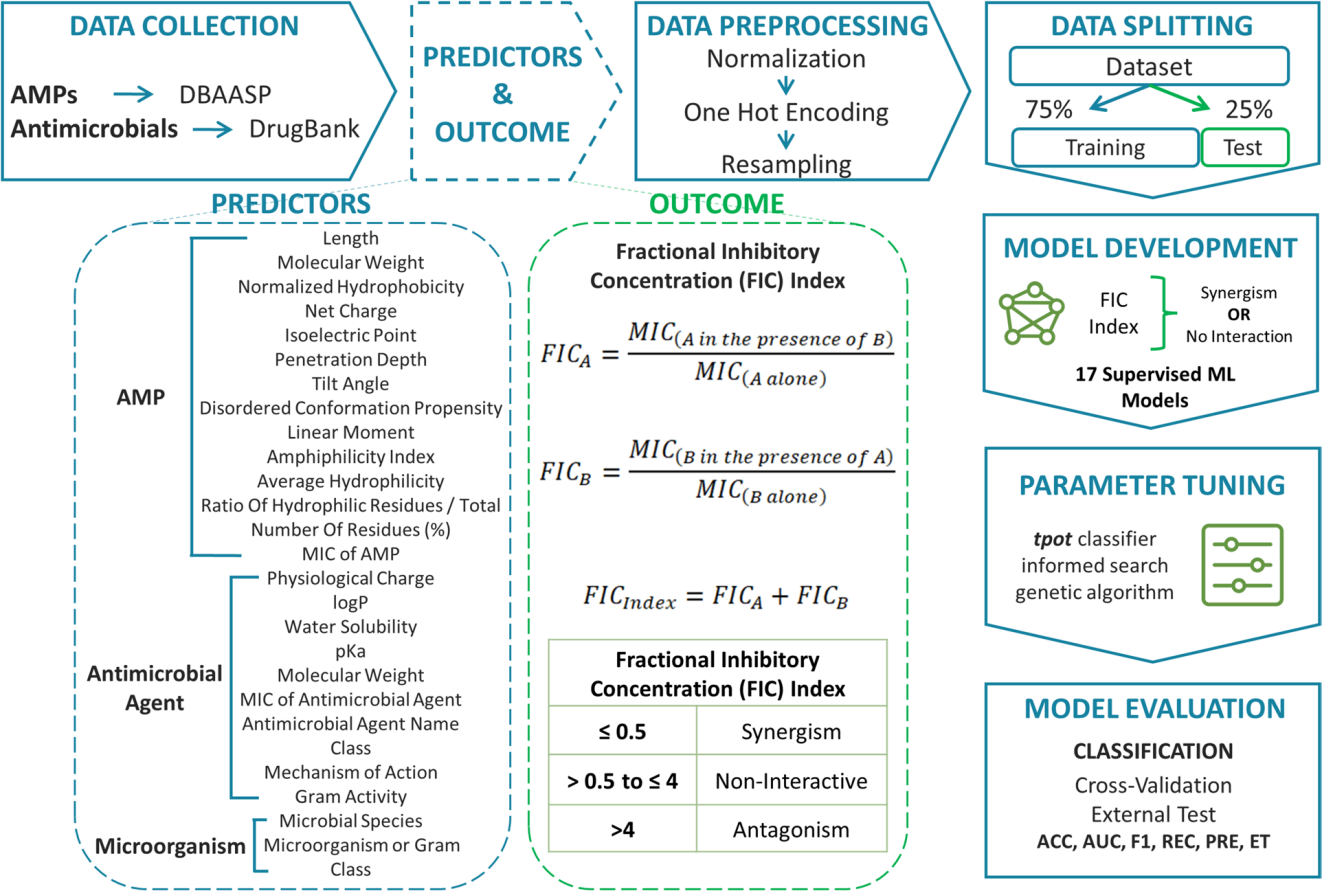
The illustration of the model development framework of the proposed study

The steps followed in this study are visually presented in Fig. 7.

### Data collection

All data were collected from the DBAASP [32] and DrugBank [34] databases. A database search was performed between March 2022 and July 2023 to find antimicrobial peptides suitable for the determined criteria and antimicrobial agents’ properties. When collecting data, sequences containing intrachain bonds, coordination bonds, and unusual amino acids were excluded in order to prevent different results arising from different interactions and structural changes between structures. The N terminal was determined to be H (without modification), and the C terminal was determined to be NH_2_^-^. As a result, 286 AMPs satisfy all the predetermined criteria. 67 AMPs remain after the exclusion of sequences that are 90% or more similar to each other. Peptides whose synergism was tested with antimicrobial agents were selected, and the properties of the antimicrobial agents of interest were collected from the DrugBank database.

### Predictors and outcome

The predictors adopted for this study are divided into three categories. The first category includes AMP characteristics such as sequence length, molecular weight, normalized hydrophobicity, net charge, IEP, penetration depth, tilt angle, disordered conformation propensity, linear moment, amphiphilicity index, average hydrophilicity, and the ratio of hydrophilic residues/total number of residues (expressed as a percentage). The second category includes antimicrobial agent characteristics such as molecular weight, class, physiological charge, logP, water solubility, pKa, and mechanism of action. Lastly, the third category includes microbial characteristics such as microbial species, gram class, and MIC. As for the outcome, the FIC index was determined to observe the synergistic effect.

Among predictors of the AMP characteristic, sequence length, the molecular weight of the sequence, normalized hydrophobicity, net charge, IEP, penetration depth, tilt angle, disordered conformation propensity, linear moment, amphiphilicity index, average hydrophilicity, the ratio of hydrophilic residues to the total number of residues, and among antimicrobial agent characteristics, the molecular weight of the antimicrobial agent, the charge of the antimicrobial agent, logP, water solubility, pKa, the activity of the peptide alone (MIC), and the activity of the antimicrobial agent alone (MIC) were numerical data. Figure S1 in Supplementary Materials presents the scatter plot of numerical predictors and outcomes. On the other hand, target species, gram stain of the target species, antimicrobial agent name, gram stain of the species in which the antimicrobial agent is active, class of the antimicrobial agent, and mechanism of action of the antimicrobial agent were nominal data.

The FIC index is commonly utilized in combination studies, and its interpretation regarding antagonism may vary among authors [50]. When the FIC index equals or falls below 0.5, it signifies a synergistic effect between the two antimicrobial agents. An FIC index ranging from 0.5 to 4 indicates a lack of substantial interaction between these antimicrobial agents. Conversely, an FIC index exceeding 4 implies antagonism between antimicrobial agents [51–53].

### Data preprocessing

As a first phase of preprocessing, data normalization was performed to improve model performance since magnitude ranges vary and may impact model optimization during training. The model’s accuracy is reduced by the extremes of the values [39]. Therefore, the more closely the values resemble one another, or the more evenly dispersed they are, the better the model will perform. With the four normalization methods, including min-max scaling, Z-score normalization, maximum absolute (max-abs) scaling, and robust data scaling [54, 55], the data were rescaled and models’ performances were compared. Figure S2 in Supplementary Materials shows the initial numerical data distribution and the modified distributions generated after four different normalization techniques. The normalization method with the highest accuracy was determined empirically and implemented for the further steps of this study.

In the second preprocessing phase of this study, feature encoding was performed. Most of the ML algorithms cannot operate with nominal data; hence, these data must be converted into numerical data [39]. The OHE method is a binary representation of the nominal data. To begin, this step requires translating the values to integer values. Later, integer values are represented in the form of binary vectors, with all values being zero except the integer index, which is labeled as 1 [56]. OHE makes nominal data representation easier and more expressive. Due to the fact that some of the predictors were nominal and had no order, these nominal data were converted to numerical data using the OHE method. Furthermore, the determined categorical output, the FIC index, was encoded for classification models gradually owing to its rank ranging from “Synergism” to “No interaction” classes. Because the “Synergism” class is superior to the other, it was encoded as “1”, whereas the“No interaction” class was encoded as “0”.

The last phase of preprocessing was resampling. If there is an imbalance in the instance numbers that constitute a class in a dataset, the expected outcomes will be affected when used as training data for ML. To fix the imbalance in the training data, resampling is commonly employed, which balances the number of observations for different classes [57]. SMOTE is a technique to increase the number of minority instances in a balanced manner in a dataset. With this method, new synthetic samples were generated from existing minority observations that were provided as input to balance the instance numbers that constitute a class in a dataset.

### Data splitting

Data splitting is a method that is widely adapted in ML. In order to train the model and test its performance, the data is split into training and test sets [56]. Random splitting algorithms pick a number of samples randomly as the training set, while the remaining samples are used as the test set [58]. In this study, the dataset was randomly split into two sets: a training set (containing 75% of the data) that was used to train the model, and a test set (containing 25% of the data) that was used to test the accuracy of the model. Moreover, data from the training set was taken for validation, and the n-repeated k-fold cross-validation method [23] was utilized in this study. Also, to ensure model generalizability, an external test set and leave-one-out scenarios [59] were adopted.

### Model development

The learning process begins with observation or data, which is then used to build a knowledge base, and then using it to detect patterns and make decisions for problems brought to it [60]. Learning is the most crucial part of this process. Based on the training set utilized and how it is interpreted for the learning process, learning can be classified into two categories: supervised and unsupervised learning [24]. Supervised learning uses labeled example data from previous experiences to predict future events with new data. The learning algorithm generates a function to anticipate output values for the given problems. Supervised learning can be divided into two categories: classification and regression. In this study, different classification techniques were utilized for the prediction of interactions between AMPs and antimicrobial agents. Models were developed using Python version 3.7.1 and *scikit-learn* version 1.0.2.

Since there is no clear consensus on model selection [39], various classification algorithms were developed, including LR [61], Linear Discriminant Analysis (LDA) [62], GPC [63], Extreme Gradient Boosting Classifier (XGBC) [64], LGBMC [41], k-nearest neighbor learning (KNN) [65], Decision Tree Classifier (DTC) [66], ETC [67], GNB [68], BNB [68], SVM [69], BC [70], AdaBoost classifier (ABC) [71], HGBC [71], RFC [72], Gradient Boosting Classifier (GBC) [71], and MLPC [73] to determine best accurate one on prediction of the FIC index. Average cross-validation accuracy scores were compared for each classifier, and the classifier with the highest accuracy score was adapted to develop the final optimized model.

### Parameter tuning

The process of determining the correct combination of hyperparameters that maximizes the performance of a model is known as hyperparameter tuning [42]. It works by running several trials in one training process. To provide the set of hyperparameter values that are most suited for the model to provide optimal results, parameter tuning was performed. A number of strategies exist in the literature for hyperparameter tuning, including random search, grid search, and informed search. A genetic algorithm is a technique for hyperparameter tuning based on the real-world concepts of genetics and informed search. The approach begins with the construction of several models, followed by the selection of the best one, the construction of other models that are similar to the best models, and the addition of randomness until the desired accuracy is achieved [42]. Genetic algorithms and informed search both employ grid and random search. The *tpot* library predicts the optimal hyperparameter values and the algorithm selects the best model based on previous iterations [74]. In this study, as an informed search approach for classification models, the *tpot* classifier was adapted with a genetic algorithm. Besides the various model parameters that were also adjusted to increase the model’s accuracy, the most accurate model was also obtained once again by parameter tuning.

### Performance evaluation

The performance of the different models was evaluated with robust metrics such as ACC, F1, REC, PRE, and Cohen’s kappa coefficient ($\kappa$). The formulas of the aforementioned metrics are explained in detail in [39]. The confusion matrix was created to visualize the correct and incorrect predictions. The ROC curve was also provided according to the TPR and FPR. Techniques for calculating a weighted score of the model’s function for all predictors in the best model are referred to as feature importance analysis. The scores simply show each feature’s contribution to model prediction ability [75]. The sum of all feature importance values is equal to 1. A higher score suggests that the specific attribute has a larger impact on the prediction model. The importance of the features was examined via feature importance analysis, and the most important features were determined. Additionally, a one-way ANOVA test [76] was performed to determine whether the classifiers’ performance was statistically significant or not.

### Supplementary Information

**Additional file 1.**

## Data Availability

Raw data were collected from the DBAASP [32] and DrugBank [34] databases. Processed data, source code, and trained models that support the findings of this study are publically available at GitHub. All collected data with their proper references are available in the online version of this paper.

## Abbreviations

ABC: AdaBoost Classifier
ACC: Accuracy
AI: Artificial Intelligence
AMP: Antimicrobial Peptide
AUC: The Area Under the Curve
BC: Bagging Classifier
BNB: Bernoulli Naïve Bayes
DT: Decision Tree
DTC: Decision Tree Classifier
ET: Elapsed Time
ETC: Extra Tree Classifier
F1: F1 Score
FIC: Fractional Inhibitory Concentration
FPR: False Positive Rate
GBC: Gradient Boosting Classifier
GNB: Gaussian Naïve Bayes
GPC: Gaussian Process Classifier
HAI: Hospital-Acquired Infections
HDP: Host Defense Peptide
HGBC: Histogram Gradient Boosting Classifier
KNN: K-Nearest Neighbor
LDA: Linear Discriminant Analysis
LGBMC: Light Gradient Boosted Machine Classifier
LR: Logistic Regression
MaxEnt: Maximum-entropy
MIC: Minimum Inhibitory Concentration
ML: Machine Learning
MLPC: Multilayer Perceptron Classifier
OHE: One Hot Encoding
PRE: Precision
REC: Recall
RF: Random Forest
RFC: Random Forest Classifier
ROC: Receiver Operating Characteristic
SVM: Support Vector Machines
SMOTE: Synthetic Minority Oversampling Technique
TPR: True Positive Rate
XGBoost: Extreme Gradient Boost
XGBC: Extreme Gradient Boosting Classifier
