# Supplementary material for: Prediction of the synergistic effect of antimicrobial peptides and antimicrobial agents via supervised machine learning
Source: BMC Biomed Eng. 2024 Jan 17;6:1. doi: 10.1186/s42490-024-00075-z (PMC10792927; doi:10.1186/s42490-024-00075-z)

**Supplementary Materials**

**Figure S1:** The scatter plot of numerical predictors and outcome (Len: Length of AMP, AMP MW: Molecular weight of AMP, Hydropho: Normalized hydrophobicity of AMP, AMP Chrg: Net charge of AMP, IEP: Isoelectric point of AMP, Penet: Penetration depth of AMP, Tilt: Tilt angle of AMP, DCP: Disordered conformation propensity of AMP, Lin Mo: Linear moment of AMP, Amph In: Amphiphilicity index of AMP, Hydrophi: Average hydrophilicity of AMP, Ratio H T: Ratio of hydrophilic residues/total for AMP, Antimic Chrg: Physiological charge of antimicrobial, logP: LogP value of antimicrobial, Wat Sol: Water solubility of antimicrobial, pKa: pKa value of antimicrobial, Antimic MW: Molecular weight of antimicrobial, AMP MIC: Activity of the peptide alone, Antimic MIC: Activity of the antimicrobial alone, FICI num: FIC index numeric).


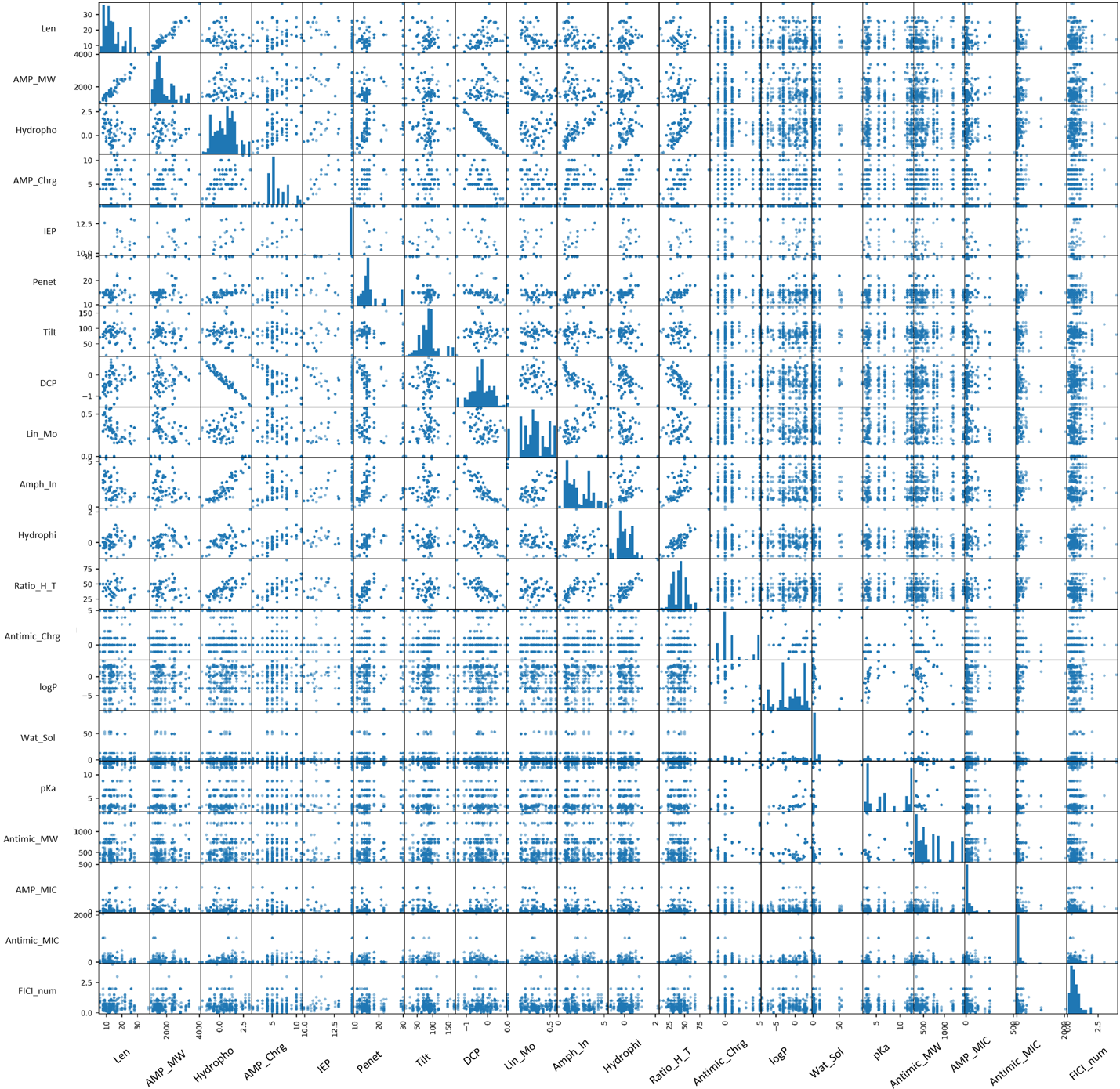


**Figure S2:** The data distribution of numerical data in the dataset for before and after the four different normalization techniques: (A) original data distribution, data distribution after (B) Z-score normalization, (C) Min-max normalization, (D) Max-absolute scaler normalization, and (E) Robust scaler normalization (Len: Length of AMP, AMP MW: Molecular weight of AMP, Hydropho: Normalized hydrophobicity of AMP, AMP Chrg: Net charge of AMP, IEP: Isoelectric point of AMP, Penet: Penetration depth of AMP, Tilt: Tilt angle of AMP, DCP: Disordered conformation propensity of AMP, Lin Mo: Linear moment of AMP, Amph In: Amphiphilicity index of AMP, Hydrophi: Average hydrophilicity of AMP, Ratio H T: Ratio of hydrophilic residues/total for AMP, Antimic Chrg: Physiological charge of antimicrobial, logP: LogP value of antimicrobial, Wat Sol: Water solubility of antimicrobial, pKa: pKa value of antimicrobial, Antimic MW: Molecular weight of antimicrobial, AMP MIC: Activity of the peptide alone, Antimic MIC: Activity of the antimicrobial alone, FICI num: FIC index numeric).


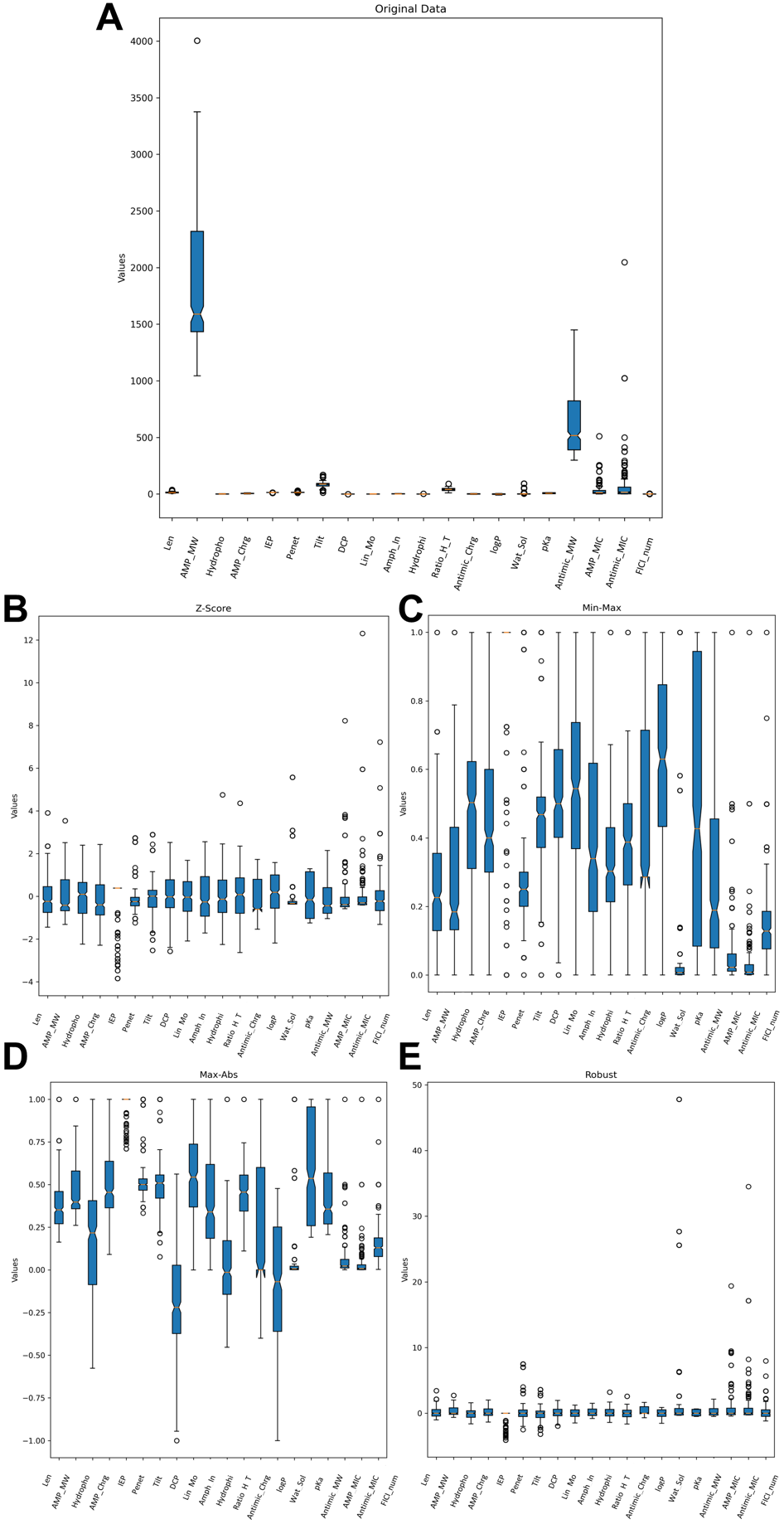

Supplement: Supplementary file 1 — Additional file 1. [file 42490_2024_75_MOESM1_ESM.zip › Supplementary Materials.docx]
